# Prevalence of Lipohypertrophy and its Associations in Insulin-Treated Diabetic Patients

**DOI:** 10.12669/pjms.39.1.6134

**Published:** 2023

**Authors:** Arsalan Nawaz, Muhammad Adnan Hasham, Maria Shireen, Mehwish Iftikhar

**Affiliations:** 1 Dr. Arsalan Nawaz, FCPS (Medicine), Assistant Professor, Department of Medicine, UCMD, University of Lahore Teaching, Hospital, Lahore, Pakistan; 2 Dr. Muhammad Adnan Hasham, FCPS (Medicine), FCPS (Endocrinology), MRCP. Assistant Professor, Department of Endocrinology & Metabolism, SIMS, Services Hospital Lahore, Pakistan; 3 Dr. Maria Shireen, FCPS (Medicine) Senior Registrar, Department of Medicine, Lahore Medical & Dental College, Lahore, Pakistan; 4 Dr. Mehwish Iftikhar, FCPS (Medicine), FCPS (Endocrinology) Assistant Professor Endocrinology, Dept of Endocrinology & Metabolism, Services Institute of Medical Sciences, Lahore, Pakistan

**Keywords:** Lipohypertrophy (LH), diabetes mellitus, Insulin

## Abstract

**Background and Objectives:**

Lipohypertrophy is a major complications of insulin therapy especially attributed to incorrect insulin technique. The objective was to determine the frequency of lipohypertrophy in diabetic population on insulin and its associations.

**Methods:**

This cross-sectional study was conducted at the Services Hospital Lahore from July 1st, 2020 to December 31st, 2020. Total of 363 diabetic patients, fulfilling the inclusion criteria were approached. The patients were examined and interviewed through a validated questionnaire. The data was stratified according to the age, gender, duration of diabetes, duration of insulin use, frequency of LH and duration since last instructions reviewed. The chi-square test was applied. Data was analyzed using SPSS 22.

**Results:**

Mean age of the study population was 49.71± 13.36 years. Majority were females (57.6%). LH was noted in 22.86% of participants (n=83). There was significant statistical difference noted (P <0.05) between different age groups as 29.7 % of patients in age group above 45 had LH as compared to 19.6% in the below 45 years age group. More females (28.2%) were having LH than the males (15.6%) with P<0.05. Also, significant difference was noted for needle reuse more than 10 times, more than two injections per day and longer duration since last instructions reviewed. No significant difference was noted between different devices for injection as well as for duration of diabetes. LH is strongly associated with hypoglycemia and hyperglycemia with P<0.05.

**Conclusion:**

A significant diabetic population on insulin has noted to have LH, and the risk is more with aged population, female gender, more injections per day and multiple time needle reuses. Risk of LH can be reduced by reinforcing education.

## INTRODUCTION

Diabetes Mellitus is a chronic metabolic disorder due to abnormal blood glucose levels. Globally it is one of the increasing non communicable diseases. In 2017, estimated global diabetic population was about 451 million which is expected to reach 693 million by 2045.^1^

Pakistan is also suffering from rising trends in diabetes mellitus. According to the second national diabetes survey of Pakistan conducted in 2016-17, one fourth of Pakistani population is suffering from diabetes mellitus.^2^,^3^ Diabetes Mellitus is associated with many micro and macrovascular complications which increases the risk of death.^4^

Mainstay of the treatment for insulin deficiency is insulin replacement, while in case of partial deficiency and resistance to insulin; oral hypoglycaemics alone or in conjunction with insulin or insulin alone are the treatment options.^5^ Early diagnosis and management of diabetes can prevent the said complications. It is recommended that wherever indicated, treatment with insulin should not be delayed as in case of marked weight loss, severe hyperglycemia and where there are contraindications to use oral hypoglycaemics.^6^,^7^

Insulin administration is associated with many complications like weight gain, hypo and hyperglycemia, needle stick injury and injection site complications including erythema, infection, abscess and most importantly lipodystrophy.^6^,^8^ Lipodystrophy can be in the form of lipohypertrophy (LH) or lipoatrophy (LA). Lipohypertrophy is a soft to firm, thickened, rubbery swelling of the soft tissue which is fibrous and relatively lacking normal vasculature while lipoatrophy is atrophy of the fatty tissues beneath the skin. LH is more commonly reported than LA.^9^,^10^ Injections into LH lead to poor glycaemic control due to variable absorption of insulin. Small needles, site rotations and avoidance of needle reuse are important to prevent LH.^10^,^11^ By an effective education program, rotation of injection site and changing the practice of needle reuse, LH can be prevented.^12^

The aim of the current study was to determine the frequency of LH among diabetic patients on insulin and its associations and risk factors. Studies related to incidence of LH are scarce in local literature. Due to limited local data available, it becomes important to determine the frequency and associations of LH so that better guidelines for the screening, treatment, and prevention of lipohypertrophy can be formulated.

## METHODS

This cross-sectional study was conducted at the Services Hospital Lahore from July 1st, 2020 to December 31st, 2020. Total 363 diabetic patients who met the inclusion criteria were enrolled in the study after providing written informed consent. Patients were examined and interviewed through a validated Questionnaire, the prime focus of which was on lipohypertrophy and its risk factors and associations. This study was approved by Institutional Review Board (Ref No.IRB/2021/838/SIMS dated 24-06-2021).

### Inclusion Criteria:

Patients of any age both genders. Diabetic patients of any type, taking insulin themselves for more than six months.

### Exclusion Criteria:

Patients who need help for injecting insulin. Patients not willing to participate.

A sample size of 329 cases is estimated with a confidence level of 95%, a margin of error of 5%, and the predicted percentage of lipohypertrophy as 31%. However total of 363 patients were enrolled in the study. For this study Non-probability consecutive sampling was utilized.

### Statistical Analysis:

Data was analyzed using SPSS version 22.0. Numerical variables, i.e., age, weight was presented as mean and standard deviation. Qualitative variables such as gender and the presence of LH were presented in the form of frequencies and percentages. Data was stratified for age, gender, duration of diabetes, number of injections per day and time since review of instructions. The chi-square test was applied to check statistical significance post-stratification.

## RESULTS

Almost all patients 98.6% were (n=359) adult, 1.2% (n=4) were adolescents i.e., below 18 years of age. Mean age of study population was 49.71±13.36 years, mean weight was 67.12±13.29 kg and mean height was 160.95±6.89 cm. Among 363 cases, 57.6 %( n=209) were females and 42.4% were males. Majority 67.5% (n=245) was above age of 45. Mean duration of diabetes was 95.18±54.49 months and mean duration of insulin use was 6.45±3.7 years. Lesser (31.7%) (n=115) were taking insulin alone. Mean number of injections was 2.45±0.8 per day. 22.9% participants (n=83) reported lump and swelling at the injection site and 100% of them have confirmed lipohypertrophy on examination. Almost all patients, 96.38% (n=80) have LH on abdomen. (Table-I). LH was more in patients who were using same syringe for more than 10 times (39.5% cases). (Table-II). Almost half of the patients (51.3%) with LH had hypoglycaemic episodes in last six months. (Table-III).

**Table-I:** Crosstabulation of LH with different parameters.

Parameter	Lipohypertrophy		P value

Total (n=363)	Yes (%)	No (%)	
***Device***			
Pen (n=31)	19.4	80.6	P >0.05(0.6)
Syringe (n=332)	23.2	76.8	
***Gender***			
Females (n=209)	28.2	71.8	P <0.05(0.005)
Males (n=154)	15.6	84.4	
Age			
Below 45 years (n=118)	29.7	70.3	P <0.05(0.03)
45 years and above (n=245)	19.6	80.4	
***Duration of diabetes***			
Up to 5 years (n=127)	22	78	P > 0.05(0.17)
6 to 10 years (n=146)	24	76	
More than 10 years (n=90)	22.2	77.8	
***Duration of insulin use***			
Up to 5 years	20.6	79.4	P > 0.05(0.36)
More than 5 years	24.6	75,4	
***Time of instructions reviewed***			
Within last 1 year (n=156)	14.1	85.9	P <0.05 (0.00)
Sometime in the last 1 to 5 years (n=191)	26.7	73.3	
Sometime in the last 5 to 10 years(n=16)	62.5	37.5
***Number of injections per day***			
Up to 2 injections per day (n=262)	19.8	80.2	P <0.05 (0.02)
More than 2 injections per day (n=101)	30.7	69.3	
***Rotation of sites***			
Yes (n=317)	15.5	84.5	P <0.05 (0.00)
No (n=46)	73.9	26.1	
***Pain at injection site***			
Yes (n=321)	24.9	75.1	P <0.05 (0.01)
No (n=42)	7.1	92.9	
***Clean site before injection***			
Yes (n=49)	38.8	61.2	P <0.05 (0.004)
No (n=314)	20.4	79.6	
***Injection into Cloth***			
Yes (n=19)	47.4	52.6	P <0.05 (0.009)
No (n=344)	21.5	78.5	
***Educator***			
Doctor	18.3	81.7	P <0.05 (0.02)
Diabetes Educator	23	77	
Pharmaceutical representative	47.4	52.6	

**Table-II:** Crosstabulation of LH with Needle reuse.

Parameter	LH %		

Needle Reuse for pen (n=31)	Yes	No
2 times (n=4)	50	50	P >0.05(0.33)
3 to 5 times (n=16)	12.5	87.5	
6 to 10 times (n=9)	22.2	77.8	
More than 10 times (n=2)	0	100	

*Needle reuse for Syringe (n=332)*	*Yes*	*No*

2 times (n=8)	0	100	P <0.05(0.00)
3 to 5 times (n=67)	20.9	79.1	
6 to 10 times (n=176)	17.6	82.4	
More than 10 times (n=81)	39.5	60.5	

**Table-III:** Crosstabulation of LH with complications.

Parameter	LH % (n-83)		
Hypoglycemia in last 6 months	Yes	No	P <0.05(0.00)
No (n=250)	10	90	
Yes (n=113)	51.3	48.7	
***Hyperglycemia in last 6 months***			
No (n=116)	14.7	85.3	P <0.05(0.01)
Yes (n=247)	26.7	73.3	

## DISCUSSION

Lipohypertrophy with insulin has been found to be a major complication of insulin therapy. Prevalence of LH has wide range in many studies ranging from 23% to 73.4%. This variability is likely due to different methods of recognizing LH.^13^,^14^

A study by Frid et al conducted in 42 countries with 13289 insulin injecting participants is the major landmark regarding worldwide insulin technique. The mean age of the study participants was 51.9±18.1 years which is greater, but comparable with our study. The mean duration since diagnosis was 13.2 years which is also higher than our study population. Also mean time since using insulin was two years higher and contrary to our study (8.7±8.9 vs 6.45±3.7 years). Prevalence of LH, reported in that study was 29%.^13^ These figures are not consistent with our data where prevalence of LH is reported to be 22.8 %.

Hauner et al reported the incidence of LH in both types of diabetes as 23.65%.^14^ A recent study in China by Ji et al reported that half of their participants (53.1%) were having LH and an Indian study by Barola et al conducted in type 1 diabetic patients reported that about two third of their study population had LH (62.6%].^15^,^16^ Another study by Pozzuoli et al whose sample was almost similar to our sample size reported the prevalence of LH to be 42.9%, while Blanco et al reported even higher prevalence (64.4%) in their data.^17^,^18^ A meta-analysis by Deng et al reported the prevalence of LH to be 38%.^19^

The wide range of prevalence may be related to using non standardized methods to diagnose LH as almost all studies relied on just examination which may be missed sometimes and examining person is also not defined in most of the studies. Study by Al Ajlouni et al in Jordan, conducted in Type-2 people with diabetes demonstrated that female gender was more associated with LH than males (28.2% vs 15.6%) which is consistent with our study. The reason for higher prevalence in females might be the less literacy rate of females in Pakistan than the males as depicted by statistics (40% vs. 69%).^20^ This study also determined that duration of diabetes and duration of insulin use are also associated with LH which is contrary to our study.^21^

Data by Gentile et al, demonstrated that risk of LH is more with old age.^22^ This is consistent with our study. Needle reuse and lack of site rotation are two important factors which leads to LH and these are endorsed by many studies. Ji et al in China found that needle reuse frequency and lack of site rotation was positively associated with LH with P value (P <0.03 and P<0.001 respectively). It was also found that two third of their participants (67.6%) with LH were rotating sites correctly while 92.3% of patients without LH were practicing correct site rotation with (p < 0.0001).^15^ Pozzuoli et al, Hauner et al and Gupta et al also determined that not rotating the site and needle reuse are strongly associated with LH.^14^,^17^,^23^

These findings are comparable with our study which shows that LH is more prevalent in patients who were not rotating the injection site and who were using needle more than 10 times. Most of the people reuse needle due to issue of cost. Frid et al also mention the cost and convenience, two major reasons for needle reuse in most of the regions around globe.^13^

Frid et al shows that least LH was found when education is given by Diabetes educator or nurses which is contrary to our study where least LH is found in patients who were taught by doctors and higher frequency was observed with diabetes educators and representatives.^13^ Variation in blood glucose leading to hypoglycemia and hyperglycemia are well known and established complications of LH. Blanco et al reported that one third (39.1%) of their patients with LH developed hypoglycemia and almost half of the cases (49.1%) with LH had hyperglycaemic episodes.^18^ This is consistent with our data. Research by Pozzuoli et al and Gupta et al also endorsed this fact that blood glucose was higher in patients who had LH and who inject into these lumps.^17^,^23^

### Limitations of the study:

It was a single centre study & almost all study participants were adults. Other limitation is that this study was conducted during the pandemic. Still, this study highlighted many aspects which were ignored in usual practice, most important of which is examination of the injection site at least biannually to pick LH early and then reinforcement of education at least once or twice a year about correct insulin technique especially regarding reuse of needles. Further larger and multicenter studies are needed to establish local guidelines for the physicians and formulating the structures education program.

## CONCLUSION

Lipohypertrophy with much higher prevalence seems to be the neglected part currently in diabetes education program. Its long-term implications are poor glycaemic control; recurrent hypoglycemia and increased financial burden are hindrance to achieve good quality of life in diabetics. This issue should be the most important part of our diabetes education program and need prompt attention from all healthcare professional and diabetes educators.

### Authors Contribution:

**AN & MAH:** Conceived, designed, data collection and did statistical analysis of manuscript.

**AN & MS:** Did manuscript writing.

**MI:** Did review & editing of manuscript and helps in statistical analysis.

**AN:** Is responsible for the accuracy of work

## References

[1] Cho N, Shaw JE, Karuranga S, Huang Y, Rocha Fernandes da JD, Ohlrogge AW, Malanda B (2018). IDF Diabetes Atlas:Global estimates of diabetes prevalence for 2017 and projections for 2045. Diabetes Res Clin.

[2] Basit A, Fawwad A, Qureshi H, Shera AS (2018). Prevalence of diabetes, pre-diabetes and associated risk factors:Second National Diabetes Survey of Pakistan (NDSP), 2016-2017. BMJ open.

[3] Aamir AH, Ul-Haq Z, Mahar SA, Qureshi FM, Ahmad I, Jawa A (2019). Diabetes Prevalence Survey of Pakistan (DPS-PAK):Prevalence of type 2 diabetes mellitus and prediabetes using HbA1c:A population-based survey from Pakistan. BMJ open.

[4] Baena-Diez JM, Penafiel J, Subirana I, Ramos R, Elosua R, Marin-Ibanez A (2016). Risk of cause-specific death in individuals with diabetes:A competing risks analysis. Diabetes Care.

[5] Arshad I, Mohsin S, Iftikhar S, Kazmi T, Nagi LF (2019). Barriers to the early initiation of Insulin therapy among diabetic patients coming to diabetic clinics of tertiary care hospitals. Pak J Med Sci.

[6] American Diabetes Association 9 (2019). Pharmacologic approaches to glycemic treatment:Standards of Medical Care in Diabetes-2019. Diabetes Care.

[7] Silver B, Ramaiya K, Andrew SB, Fredrick O, Bajaj S, Kalra S, Charlotte BM, Claudine K, Makhoba A (2018). EADSG guidelines:insulin therapy in diabetes. Diabetes Ther.

[8] Borro M, Murdaca G, Monachesi M, Negrini S (2021). Unexpected adverse event of insulin therapy in diabetes mellitus. Acta Diabetol.

[9] Nagesh VS, Kalra S (2015). Recent Advances In Endocrinology. Recent Advances In Endocrinology. J Pak Med Assoc.

[10] Blanco M, Hernández MT, Strauss KW, Amaya M (2013). Prevalence and risk factors of lipohypertrophy in insulin-injecting patients with diabetes. Diabetes Metab J.

[11] Heinemann L (2010). Insulin absorption from lipodystrophic areas:A (neglected) source of trouble for insulin therapy?. J Diabetes Sci Technol.

[12] Kalra S, Kumar A, Gupta Y (2016). Prevention of lipohypertrophy. J Pak Med Assoc [Internet].

[13] Frid AH, Hirsch LJ, Menchior AR, Morel DR, Strauss KW (2016). Worldwide injection technique questionnaire study:Injecting complications and the role of the professional. Mayo Clin Proc.

[14] Hauner H, Stockamp B, Haastert B (1996). Prevalence of lipohypertrophy in insulin-treated diabetic patients and predisposing factors. Exp Clin Endocrinol Diabetes.

[15] Ji L, Sun Z, Li Q, Qin G, Wei Z, Liu J, Chandran AB, Hirsch LJ (2017). Lipohypertrophy in China:prevalence, risk factors, insulin consumption, and clinical impact. Diabetes Technol Ther.

[16] Barola A, Tiwari P, Bhansali A, Grover S, Dayal D (2018). Insulin-related lipohypertrophy:lipogenic action or tissue trauma?. Front Endocrinol.

[17] Pozzuoli GM, Laudato M, Barone M, Crisci F, Pozzuoli B (2018). Errors in insulin treatment management and risk of lipohypertrophy. Acta diabetologica.

[18] Blanco M, Hernandez MT, Strauss KW, Amaya M (2013). Prevalence and risk factors of lipohypertrophy in insulin-injecting patients with diabetes. Diabetes Metabol.

[19] Deng N, Zhang X, Zhao F, Wang Y, He H (2018). Prevalence of lipohypertrophy in insulin treated diabetes patients:A systematic review and Meta Analysis. J Diabetes Investig.

[20] Rehman A, Jingdong L, Hussain I (2015). The province-wise literacy rate in Pakistan and its impact on the economy. Pacific Science Review B:Humanit Soc Sci.

[21] Mo'men Al, Ajlouni MA, Batieha A, Ajlouni K (2015). Prevalence of lipohypertrophy and associated risk factors in insulin-treated patients with type 2 diabetes mellitus. Int J Endocrinol Metab.

[22] Gentile S, Guarino G, Della Corte T, Marino G, Fusco A, Corigliano G Lipohypertrophy in Elderly Insulin-Treated Patients with Type 2 Diabetes. Diabetes Ther.

[23] Gupta SS, Gupta KS, Gathe SS, Bamrah P, Gupta SS (2018). Clinical implications of lipohypertrophy among people with type 1 diabetes in India. Diabetes Technol Ther.

